# Detection and Assessment of the Distribution of Infectious Agents in Juvenile Fraser River Sockeye Salmon, Canada, in 2012 and 2013

**DOI:** 10.3389/fmicb.2018.03221

**Published:** 2018-12-21

**Authors:** Omid Nekouei, Raphael Vanderstichel, Tobi Ming, Karia H. Kaukinen, Krishna Thakur, Amy Tabata, Emilie Laurin, Strahan Tucker, Terry D. Beacham, Kristina M. Miller

**Affiliations:** ^1^Department of Health Management, University of Prince Edward Island, Charlottetown, PE, Canada; ^2^Pacific Biological Station, Fisheries and Oceans Canada, Nanaimo, BC, Canada; ^3^Department of Forest & Conservation Sciences, The University of British Columbia, Vancouver, BC, Canada

**Keywords:** Sockeye salmon, Fraser River, infectious agent, prevalence, high-throughput genomic screening

## Abstract

Infectious diseases may contribute to declines in Fraser River Sockeye salmon (*Oncorhynchus nerka*) stocks, but a clear knowledge gap exists around which infectious agents and diseases are important. This study was conducted to: (1) determine the presence and prevalence of 46 infectious agents in juvenile Fraser River Sockeye salmon, and (2) evaluate spatial patterns in prevalence and burden over initial seaward migration, contrasting patterns between 2 years of average and poor productivity. In total, 2,006 out-migrating Sockeye salmon were collected from four regions along their migration trajectory in British Columbia, in 2012 and 2013. High-throughput microfluidics quantitative PCR was employed for simultaneous quantitation of 46 different infectious agents. Twenty-six agents were detected at least once, including nine with prevalence >5%. *Candidatus Brachiomonas cysticola, Myxobolus arcticus*, and Pacific salmon parvovirus were the most prevalent agents. Infectious agent diversity and burden increased consistently upon smolts entry into the ocean, but they did not substantially change afterwards. Notably, both freshwater- and saltwater-transmitted agents were more prevalent in 2013 than in 2012, leading to an overall higher infection burden in the first two sampling regions. A reduction in the prevalence of two agents, erythrocytic necrosis virus and *Paraneuclospora theridion*, was observed between regions 2 and 3, which was speculated to be associated with mortality during the 1st month at sea. The most prevalent infectious agents were all naturally occurring. In a small number of samples (0.9%), seven agents were only detected around and after salmon farming regions, including four important pathogens: piscine orthoreovirus, *Piscirickettsia salmonis, Tenacibaculum maritimum*, and *Moritella viscosa*. As the first synoptic survey of infectious agents in juvenile Sockeye salmon in British Columbia, this study provides the necessary baseline for further research on the most prevalent infectious agents and their potential pathogenicity, which may adversely affect the productivity of valuable Sockeye salmon stocks. In addition, our findings are informative to the decision makers involved in conservation programs.

## Introduction

Sockeye salmon (*Oncorhynchus nerka*) have symbolic, cultural, recreational, and economic significance to the residents of the Pacific North, especially in Canada (Smith et al., 1987; Groot and Margolis, 1991). Sockeye is the third most abundant of the seven species of Pacific salmon, after Pink (*O. gorbuscha*) and Chum (*O. keta*). They often travel long distances in freshwater watersheds to reach diverse spawning habitats, to which they are highly adapted (Groot and Margolis, 1991).

The Fraser River, located in the Canadian province of British Columbia (BC), supports the largest abundance of Sockeye salmon in the world for a single river (Cohen, 2012). This river is 1,600 km long, with a vast watershed of 223,000 km^2^ (Groot and Margolis, 1991; Cohen, 2012). In the Fraser River system, approximately, 90 Sockeye salmon spawning populations have been identified (Gable and Cox, 1993; Thomson et al., 2012). They typically have a 4-year lifespan and a lake-type life history, in which fry emerge from the gravel in a river and migrate to a nursery lake, where all but one of the 90 stocks (Harrison River) rear for 1 or 2 years before entering the ocean (Beamish et al., 2016). Sockeye smolts enter the Strait of Georgia over a 2-month period from late March to late May (Preikshot et al., 2012) and move northward through the Strait of Georgia over 5–6 weeks from mid-May to mid-July (Beamish et al., 2012; Thomson et al., 2012).

In recent decades, the majority of Fraser River Sockeye salmon stocks have shown a substantial decline in abundance and productivity (Beamish et al., 2012; Cohen, 2012; Peterman et al., 2012). Despite significant reductions in harvest, productivity and abundance of returning adults consistently decreased from the early 1990s to 2009; the 2009 returns were the lowest since 1947 (Peterman et al., 2012). This issue led to the establishment of a judicial inquiry in November 2009 (herein, Cohen Commission) to investigate the potential reasons for this decline. After considerable expert testimony, the final report for the Cohen Commission of Inquiry highlighted the role of the early marine environment as the location where year-class strength is likely determined. Factors such as climate change impacts on temperature and prey availability, infectious diseases, predation, and the interplay amongst these factors were identified as main potential contributors to declines (Cohen, 2012; Miller et al., 2014). Importantly, infectious diseases were included based on the theoretical risk that they could pose on survival at the population level. It was recognized that there simply was not enough information on what pathogens and diseases may impact Sockeye salmon in the ocean to provide direct knowledge of specific disease risks. Filling in this knowledge gap was the primary motivation for our research.

Infectious diseases can disrupt salmon’s normal behavior and physiological performance (e.g., swimming and visual acuity), immunological function, feeding and growth, and can cause mortality in severe cases (Costello, 2006; Miller et al., 2014). Our current understanding of infectious diseases in salmon mainly arises from studies conducted on cultured fish (i.e., hatcheries and ocean net pens), where we can observe and assess clinical diseases and mortality. However, we expect that the distribution and impacts of infections in wild migratory fish may differ from those in cultured fish. In salmon aquaculture, acute infectious diseases are likely to be more impactful due to high rearing densities of susceptible fish, leading to high levels of fish with clinical signs and/or mortality in short periods of time. Alternately, in low-density migratory populations, pathogens causing acute diseases may not have sufficient time (before mortality) to spread; hence, while they may directly cause mortality in some individuals, they may be less likely to have population-level impacts (Bakke and Harris, 1998; Miller et al., 2014). Instead, we expect that chronic infections likely play a more important role in the overall survival and performance of wild salmon, given the longer duration for transmission and potential indirect effects on behavior and performance (Bakke and Harris, 1998; Miller et al., 2014, 2017).

In addition to natural sources of infection, the potential spillover of pathogens from salmon farms to sympatric wild fish has been a contentious issue since the onset of aquaculture in BC (Nekouei et al., 2018). For instance, two infectious agents have recently drawn a lot of media attention, parasitic salmon lice (*Lepeophtheirus salmonis*) and piscine orthoreovirus (PRV) (Krkošek, 2010; Torrissen et al., 2013; Marty et al., 2015; Di Cicco et al., 2017; Purcell et al., 2017).

The knowledge gap regarding infectious diseases that can adversely affect the performance and survival of wild Pacific salmon is largely due to the unobservable nature of the clinical signs and mortality along their migration routes. Out-migrating juveniles are particularly vulnerable to environmental stressors during their early marine life, including many infectious agents they may potentially acquire along the way, and >90% of them may die in this limited period (Beamish and Mahnken, 2001; Mckinnell et al., 2014). Although there have been a number of monitoring studies focused on the distribution of readily observable parasites in wild Pacific salmon, most were not specific to Sockeye salmon, and only few studies included viral and bacterial pathogens (Kent, 2011). Moreover, there are numerous infectious agents that have been identified in association with emerging diseases around the world that have never been assessed in Sockeye salmon.

To address the outlined knowledge gaps and establish a baseline for common infectious agents in juvenile Sockeye salmon, we conducted the present study to: (1) determine the presence, prevalence, and burden of 46 important infectious agents in juvenile Fraser River Sockeye salmon using high-throughput microfluidics qPCR, and (2) evaluate spatiotemporal shifts in the prevalence and burden of these agents, contrasting patterns between 2 years of average (2012, out-migration) and very poor productivity (2013, out-migration).

## Materials and Methods

### Sample Collection

**FIGURE 1:**
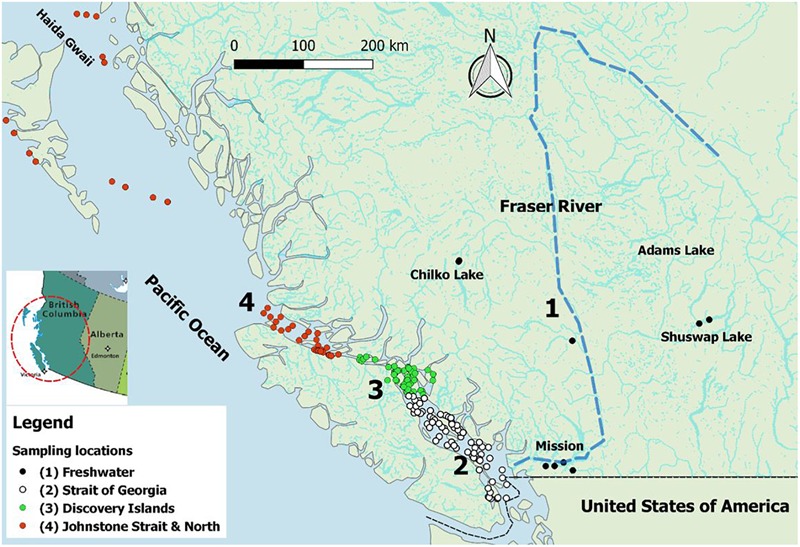
Map of British Columbia (BC), Canada, illustrating the Fraser River watershed (the main stream is dashed) and the four sampling regions from which 2,006 juvenile Fraser River Sockeye smolts were collected for this study. The out-migration route is from ‘Region 1’ to ‘Region 4.’ This map was created in QGIS v2.18.13 (http://www.qgis.org).

Sampling of juvenile Sockeye salmon was carried out in the spring and summer of 2012 and 2013, concurrent with the out-migration window for the majority of Fraser River Sockeye populations (i.e., April–July). In freshwater, sampling was carried out using beach seining within natal lakes or dip netting at smolt fences. In the lower Fraser River, sampling was conducted via a rotary screw trap in Mission (Figure 1). In the marine environment, fish were collected through multiple sampling programs. Trawl samples were collected from the Canadian Coast Guard vessel, WE Ricker, or smaller contracted vessels; and purse seine collections were conducted from fishing vessels contracted by Fisheries and Oceans Canada (DFO). On the trawl vessels, fish were kept on ice and processed within 30 min of being brought on-deck. On purse seine vessels, samples were placed in a holding tank on deck until processing. Upon sampling, live fish were euthanized in MS-222, assigned a unique identification code, and five tissues (gill, whole brain, heart, liver, and kidney) were sampled aseptically from each fish and preserved in RNAlater (Qiagen, MD, United States). Tissue samples were kept at 4°C overnight, and transferred to -20°C for short-term or -80°C for long-term storage until further processing. Additional fish collected at sea and all freshwater collections were frozen at -80°C until further processing in the laboratory. Fin clips were also taken at the time of collection and preserved in 95% ethanol for genetic stock identification. Genetic stock identification of individual fish was performed using microsatellite analysis of DNA from fin clips, according to Beacham et al. (2010, 2014). All samples not collected within natal rearing areas underwent genetic testing. Only Fraser River-origin fish were retained for analyses. Overall, 2,289 Sockeye smolts were available from along the migratory trajectory from freshwater natal rearing areas within the Fraser River watershed, into the marine environment, and over migration between the Strait of Georgia and Haida Gwaii (Figure 1).

### Laboratory Analysis

Sampled tissues were individually homogenized in TRI-reagent (Ambion Inc., Austin, TX, United States) before extraction. ‘1-bromo-3-chloropropane’ was added to the homogenate, which was then centrifuged to separate aqueous and organic phases. Equal volumes of the aqueous phase for each tissue within a fish were combined and placed into a 96-well plate for RNA extraction. Total RNA was extracted from each sample using the MagMax-96 for Microarrays Total RNA Isolation Kit (Ambion Inc.) on a Biomek NXP automated liquid-handling instrument using the ‘spin method’ protocol. RNA quantity and quality were assessed by measuring the A260/280/230 on a Beckman Coulter DTX 880 Multimode Detector (Brea, CA, United States), and samples diluted to 62.5 ng/μL. RNA (1 μg) was reverse-transcribed to cDNA using SuperScript VILO MasterMix (Invitrogen, Carlsbad, CA, United States) as per the manufacturer’s protocol.

DNA was isolated from the organic/interphase portion of TRI-reagent using a high salt TNES-urea buffer (Asahida et al., 1996), followed by the BioSprint 96 DNA extraction kit on a BioSprint 96 workstation (Qiagen, MD, United States). DNA quantity and quality were assessed by measuring the A260/280 on a Beckman Coulter DTX 880 Multimode Detector (Brea, CA, United States), and normalized to 62.5 ng/μL. Equal volumes of DNA and cDNA were combined and used as template for the qPCR assays.

Combined multi-tissue samples of DNA/cDNA for each fish were assessed for the presence and load (abundance) of our infectious agents of interest. Assays were run in duplicate on the Fluidigm BioMark^TM^ HD platform (Fluidigm, South San Francisco). This platform is able to assess 96 assays on 96 samples at once (9,216 individual reaction chambers). Our 96 assays in each run included 47 agents in duplicate, one housekeeping gene (endogenous control), and one negative assay control. Forty five of the 46 infectious agents of interest were known or suspected to cause diseases in salmon worldwide (Miller et al., 2014); *Kudoa thyrsites* is not a pathogen but of economic importance. The infectious agents were 11 viruses (two strains for infectious salmon anemia virus [ISAV]), 14 bacteria and 21 parasites (Table 1).

**Table 1:** Forty-six infectious agents (including two strains for ‘isav’) tested on 2,006 out-migrating Fraser River Sockeye salmon in 2012 and 2013, along with their overall prevalences.

Agent	Type^a^	Abbreviation	*n*	Positive	Prevalence (%)^b^
(1) *Candidatus Brachiomonas cysticola*	B	c_b_cys	1,996	1,800	90.18
(2) *Myxobolus arcticus*	P	my_arc	1,970	1,180	59.90
(3) Pacific salmon parvovirus	V	pspv	1,975	1,119	56.66
(4) *Paraneuclospora theridion*	P	pa_ther	1,982	622	31.38
(5) *Parvicapsula minibicornis*	P	pa_min	1,960	503	25.66
(6) *Parvicapsula kabatai*	P	pa_kab	1,992	320	16.06
(7) Erythrocytic necrosis virus	V	env	1,991	210	10.55
(8) *Ichthyophthirius multifiliis*	P	ic_mul	1,888	156	8.26
(9) Gill chlamydia	B	sch	1,989	135	6.79
(10) *Parvicapsula pseudobranchiocola*	P	pa_pse	1,978	58	2.93
(11) *Ichthyophonus hoferi*	P	ic_hof	1,985	53	2.67
(12) *Flavobacterium psychrophilum*	B	fl_psy	1,973	35	1.77
(13) *Ceratomyxa shasta*	P	ce_sha	1,998	32	1.60
(14) Rickettsia-like organism	B	rlo	1,993	27	1.35
(15) *Dermocystidium salmonis*	P	de_sal	2,004	27	1.35
(16) *Sphaerothecum destructuens*	P	sp_des	2,005	21	1.05
(17) *Tetracapsuloides bryosalmonae*	P	te_bry	2,006	19	0.95
(18) *Loma salmonae*	P	lo_sal	1,996	16	0.80
(19) *Kudoa thyrsites*	P	ku_thy	2,006	6^∗^	0.30
(20) *Tenacibaculum maritimum*	B	te_mar	2,006	5^∗^	0.25
(21) *Facilispora margolisi*	P	fa_mar	1,996	2^∗^	0.10
(22) *Moritella viscosa*	B	mo_vis	2,006	2^∗^	0.10
(23) *Crybtobia salmonistica*	P	cr_sal	2,006	2	0.10
(24) Piscine OrthoReovirus	V	prv	1,929	1^∗^	0.05
(25) *Piscirickettsia salmonis*	B	pisck_sal	2,005	1^∗^	0.05
(26) *Nanophyetus salmincola*	P	na_sal	2,006	1^∗^	0.05
(27) *Aeromonas hydrophila*	B	ae_hyd	2,006	0	0.00
(28) *Aeromonas salmonicida*	B	ae_sal	2,006	0	0.00
(29) *Gyrodactylus salaris*	P	gy_sal	2,006	0	0.00
(30) Infectious hematopoietic necrosis virus	V	ihnv	2,006	0	0.00
(31) Infectious pancreatic necrosis virus	V	ipnv	2,005	0	0.00
(32) Infectious salmon anemia virus (S7)	V	isav7	1,999	0	0.00
(33) Infectious salmon anemia virus (S8)	V	isav8	2,006	0	0.00
(34) *Myxobolus insidiosus*	P	my_ins	2,005	0	0.00
(35) *Neoparamoeba perurans*	P	ne_per	1,929	0	0.00
(36) *Nucleaospora salmonis*	P	nu_sal	2,000	0	0.00
(37) Salmonid herpesvirus	V	omv	2,006	0	0.00
(38) *Piscichlamydia salmonis*	B	pch_sal	2,006	0	0.00
(39) Piscine myocarditis virus	V	pmcv	2,004	0	0.00
(40) *Renibacterium salmoninarum*	B	re_sal	2,006	0	0.00
(41) Salmon alpha virus 1/2/3	V	sav	2,003	0	0.00
(42) *Spironucleus salmonicida*	P	sp_sal	2,006	0	0.00
(43) Viral encephalopathy and retinopathy virus	V	ver	2,006	0	0.00
(44) Viral hemorrhagic septicemia virus	V	vhsv	2,005	0	0.00
(45) *Vibrio anguillarum*	B	vi_ang	2,006	0	0.00
(46) *Vibrio salmonicida*	B	vi_sal	2,006	0	0.00
(47) *Yersinia ruckeri*	B	ye_ruc_gln	2,005	0	0.00

*^*a*^Type of agent: B, bacterium; V, virus; P, parasite.*

*^*b*^Overall ‘prevalence’ was defined as the number of test-positive samples divided by the total number of samples tested for each given infectious agent with conclusive results (i.e., positive/n). The 26 detected agents are presented in decreasing order of their prevalence. Agents first detected around and after salmon farming regions are shown with ‘^∗^’ under the ‘Positive’ column*.

Each run included 80 samples and 16 controls, including 5 serial dilutions of artificial positive constructs (APC) (for quantification of all infectious agents) as well as positive and negative processing controls. Negative controls included three negative processing controls for RNA/DNA extraction, two no-template controls for template enrichment (described below), two cDNA (no reverse transcriptase) controls, and two no-template controls for PCR. Positive controls included duplicates of a pooled sample from the cDNA/DNA for all fish used in the study, an endogenous reference gene to assess RNA quality, and five serial dilutions of APC clones for all assays to both assess assay integrity and to calculate the copy number of each detected agent; APC clones were loaded last to minimize the potential for contamination. The APC clones were synthesized and cloned sequences of the amplicon for each assay contained an “extra” probe sequence so that potential contamination of high concentration APCs in sample wells could be identified.

As the BioMark microfluidics platform uses very small assay volumes (7 nL), pre-amplification of assays is required to optimize sensitivity of detection. Primer pairs for each of the 48 assays were combined with TaqMan Preamp MasterMix (Applied Biosystems, Foster City, CA, United States) for a final concentration of 50 nM in a 5 μL reaction, and run through 14 cycles of amplification, according to the BioMark protocol. ExoSAP-IT (Affymetrix, Santa Clara, CA, United States) was used to remove unincorporated primers before the samples were diluted 1:5 in DNA Suspension Buffer (Teknova, Hollister, CA, United States).

Artificial positive constructs (APC) were created from each assay region’s sequence, with an additional common probe sequence added to each construct, allowing for detection of vector contamination (see Miller et al., 2016). Five serial dilutions of combined, known concentration, APC clones were run on each dynamic array for calculation of assay efficiency and copy number.

A 5 μL sample mix was prepared for each pre-amplified sample with TaqMan Universal Master Mix (Life Technologies), GE Sample Loading Reagent (Fluidigm), and a 5 μL aliquot of assay mix was prepared containing 10 μM primers and 3 μM probes for each separate TaqMan assay. An IFC controller HX pressurized and mixed the assays and samples from their individual inlets on the chip. PCR conditions were: 50°C for 2 min, 95°C for 10 min, followed by 40 cycles of 95°C for 15 s, and 60°C for 1 min on the BioMark Dynamic Array.

Cycle threshold (Ct) was determined using the Biomark Real-Time PCR analysis software. Visual evaluation identified abnormal curve shapes, presence of APC contamination, and correlation of replicates.

For each infectious agent assay in this study, the analytically validated limit of detection (Miller et al., 2016) was applied to the average Ct values (from duplicate qPCR assays) to categorize the test results as either positive or negative. These dichotomized results were further used in the calculation of prevalence and other statistical analyses. The limit of detection is defined as the estimated Ct number under which a given assay is expected to provide true positive results in 95% of the times (Miller et al., 2016). If an infectious agent assay detected a Ct signal in only one of the two replicates, the sample was considered ‘inconclusive’ for that assay and treated as a missing value in our final analyses. All technical details, validation procedures, and performance of our laboratory methodology have been reported in Miller et al. (2016).

### Statistical Analysis

Of 2,289 collected Sockeye smolts, 2,062 belonged to 40 different stocks from the Fraser River system (Supplementary Table S2). Five observations were dropped due to numerous missing values. For the final analyses, fish belonging to ‘Harrison’ stock (*n* = 51) were removed because of their distinct life history and out-migration pattern (Beacham et al., 2014). The final dataset included 2,006 out-migrating Fraser River Sockeye smolts. All statistical analyses were carried out in Stata v15.1 (StataCorp, College Station, TX, United States).

To evaluate the spatial distribution of fish in our study, sampling locations were categorized into four main regions, representing both the distinct environments encountered along the migration path of out-migrating smolts and breakpoints before, during, and after potential interaction of smolts with salmon farms (hereafter, sampling regions): (1) freshwater (along the Fraser River system), (2) Strait of Georgia (before exposure to salmon farms), (3) Discovery Islands area (the first substantial exposure to salmon farms), and (4) Johnstone Strait and northern areas (continued exposure to salmon farms, but 2+ weeks post initial exposure through the Discovery Islands and Broughton region). Figure 1 displays the localization of captured fish, colored by the four defined regions along the coast of BC. This map was created using coordinates of the sampling locations in QGIS v2.18.13^1^.

The term ‘prevalence’ for each infectious agent was defined as the number of test-positive fish divided by the total number of samples tested with conclusive results for that agent in the relevant strata in space and time. All of the 46 infectious agents (two assays for ISAV) and their overall prevalence of detection are reported in Table 1.

The term ‘load’ for each detected infectious agent was computed as log_10_ (RNA copy number + 1) from the amplification curve of the serially diluted APC clones. Given that our analyses combined cDNA and genomic DNA, duplications could occur between transcriptional and nucleic copies, and multiple copies may exist of some ribosomal genes being assayed. As such, we utilize copy number merely as a method to reflect the relative abundance among samples within an assay and should, therefore, not be extrapolated to directly compare genome copy numbers between agents.

The term ‘diversity’ for each sampled fish was defined as the sum of all infectious agents detected from that sample (i.e., the number of co-detections per fish). A Poisson regression model was built to evaluate the potential association between sampling region, year, and their interaction (representing spatiotemporal variations), on the diversity of infectious agents (the outcome of interest being the number of co-detections).

The term ‘relative infection burden’ (RIB) is a composite metric of multiple infectious agent burden using qPCR data, which was calculated from the following formula:

$$\sum_{i\in m}^{m}\frac{\text{Li}}{\text{Lmaxi}}$$

Where for a given fish, RNA copy number of the i’th positively detected infectious agent (*Li*) is divided by the maximum RNA copy number within the population for the i’th infectious agent (*Lmaxi*), and then summed across all detected agents from that fish (Teffer et al., 2018). A linear regression model was built to evaluate the potential association between sampling region, year, and their interaction, on the relative infection burden (the outcome of interest: log_10_-RIB).

After determining the overall prevalence of all infectious agents, those with a prevalence > 5% (hereafter, the common agents = the top nine agents in Table 1) were selected for further spatiotemporal statistical analyses. The reason for this selection was to avoid zero counts and/or extremely unbalanced distributions of test-positive samples at the four regional levels. These analyses are described under the following two sub-sections: (1) prevalences of the common agents and their corresponding logistic regression models, and (2) loads of the common agents in test-positive samples and their corresponding linear regression models.

### Prevalences and Logistic Regression Models

Distributions of the prevalences of common agents by sampling region and year were calculated and plotted. To evaluate the potential associations between sampling region, year, and their interaction, on the prevalence of common infectious agents, logistic regression models were built. In these analyses, presence or absence of a particular agent within a sample served as the outcome of interest.

### Loads and Linear Regression Models

Distributions of the loads of common agents in test-positive samples by sampling region and year were calculated and plotted. The potential associations between sampling region, year, and their interaction on the load of common infectious agents were evaluated using linear regression models. Diagnostics for all models in this study were evaluated to ensure that the respective models’ assumptions (Dohoo et al., 2009) were satisfied, and Bonferroni corrections were applied to the significance level of 0.05 in multiple comparisons (i.e., for comparing the outcomes of interest at the different levels and combinations of predictors in the models).

## Results

Overall, 645 and 1,361 Fraser River Sockeye smolts were analyzed from the defined sampling regions in 2012 and 2013, respectively. Of 2,006 samples, 44.7% were from freshwater (Region 1) and 55.3% from saltwater over the other three regions. The frequency distribution of the sampled fish by sampling region and year is presented in Table 2. For 1,717 of the collected fish, fork-length measurements were available, with an overall mean ± SD of 93.5 ± 22.4 mm. Supplementary Table S1 shows the fork-length distribution of the study fish by sampling region and year.

**Table 2:** Frequency distribution of the 2,006 sampled out-migrating Fraser River Sockeye salmon by study region and year.

Region	2012	2013	Total (%)
(1) Freshwater	323	573	896 (44.7)
(2) Strait of Georgia	114	238	352 (17.6)
(3) Discovery Islands	139	299	438 (21.8)
(4) Johnstone Strait and north	69	251	320 (15.9)
Total (%)	645 (32.1)	1,361 (67.9)	2,006 (100)

Based on the genetic stock identification analyses, the fish belonged to 40 different stocks in the Fraser River system. The frequency distribution of the 2,006 fish by the stock of origin is reported in Supplementary Table S2. More than half of the samples came from three major stocks: 35% originated from the Chilko stock, which, for management purposes, serves as the indicator stock for all Fraser River Sockeye salmon; and Lower Adams and Lower Shuswap had the second and third highest frequencies, with 9 and 7.6%, respectively. The rest of the 37 stocks were represented by fewer than 100 fish, with extremely unbalanced distributions across the regions and years (Supplementary Table S2).

Of the 46 infectious agents investigated within each individual fish sample, 26 agents were detected at least once (Table 1). Nine infectious agents had an overall prevalence > 5% (i.e., the common agents). *Candidatus Brachiomonas cysticola* (c_b_cys) was observed at the highest overall prevalence (90.2%), detected in 1,800 out of 1,996 samples with conclusive results, followed by *Myxobolus arcticus* (my_arc; 59.9%) and Pacific salmon parvovirus (pspv; 56.7%). Twenty infectious agents were not detected at all in our samples. To improve clarity in visualization, only abbreviations for the common infectious agents are used in the figures (see key in Table 1).

**FIGURE 2:**
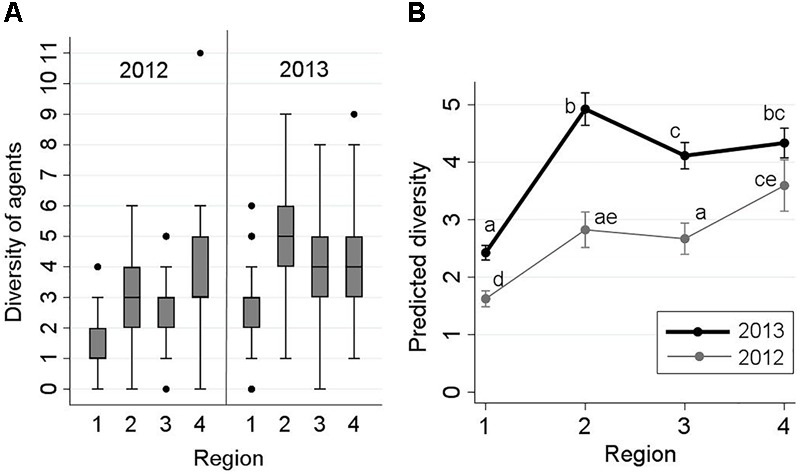
Frequency distribution of the diversity of infectious agents detected in 2,006 Fraser River juvenile Sockeye salmon by sampling region and year (left side: **A**), and the interaction plot for the results of the Poisson regression model, indicating predicted number of detected infectious agents (*Y*-axis) from fish by sampling region (*X*-axis) and year (right side: **B**). Sampling regions: (1) Freshwater, (2) Strait of Georgia, (3) Discovery Islands, and (4) Johnstone Strait and north. Statistical comparisons among the predicted diversity of these regions are presented by small letters on the graph (Bonferroni groups). Small letters in **(B)** represent statistically significant differences (at α = 0.05) between the points (unique combinations of ‘region’ and ‘year’), obtained from pairwise comparisons using Bonferroni adjustments. Points that share, at least, a letter are not statistically different (*P* > 0.05), and points with no letters in common have statistically significant differences in the predicted diversity (*P* ≤ 0.05).

The frequency distribution of the diversities of detected infectious agents (co-detections) from each fish by sampling region and year is presented in Figure 2A. Diversity ranged between 0 and 11. The maximum of 11 agents was detected from only one fish taken from ‘Region 4’ in 2012. Approximately, 90% of the samples had a diversity of ≤5. The results of the respective Poisson model (indicating changes in the predicted number of detected agents by study region and year) are illustrated using an interaction plot in Figure 2B. The interaction between region and year was statistically significant (*P* = 0.001). According to Figure 2, diversity of agents from freshwater to saltwater (Strait of Georgia) significantly increased, but changes in the three regions within saltwater were not substantial in either 2012 or 2013. Diversities in all four regions were greater in 2013 than their corresponding points in 2012 (Figure 2B). For instance, the median diversity in ‘Region 2’ was 3 in 2012, and 5 in 2013 (Figure 2A).

**FIGURE 3:**
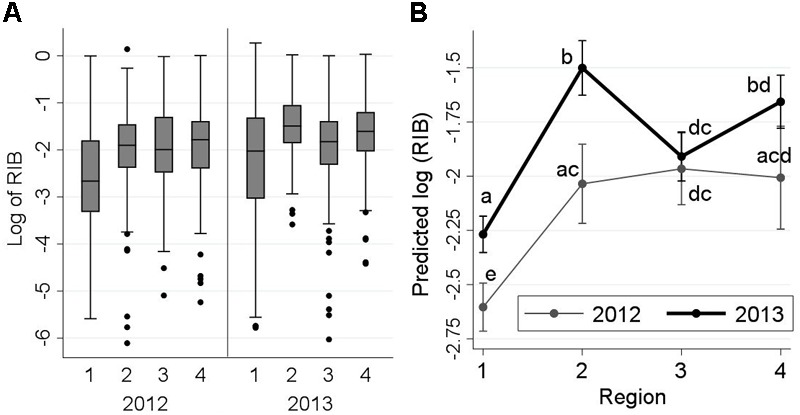
Frequency distribution of log_10_-relative infection burden (RIB) of all detected infectious agents from 2,006 Fraser River juvenile Sockeye salmon by sampling region and year (left side: **A**), and the interaction plot for the results of the linear regression model (right side: **B**), indicating predicted log_10_-RIB (*Y*-axis) for the fish by year and sampling region (*X*-axis). Sampling regions: (1) Freshwater, (2) Strait of Georgia, (3) Discovery Islands, and (4) Johnstone Strait and north. Statistical comparisons among the predicted log_10_-RIB of these regions are presented by small letters on the graph. Small letters in **(B)** represent statistically significant differences (at α = 0.05) between the points (unique combinations of ‘region’ and ‘year’), obtained from pairwise comparisons using Bonferroni adjustments. Points that share, at least, a letter are not statistically different (*P* > 0.05), and points with no letters in common have statistically significant differences in the predicted RIB (*P* ≤ 0.05).

Relative infection burden ranged between 0 and 3.51, with an extremely right-skewed distribution; therefore, a logarithmic transformation was applied to RIB values. The frequency distribution of log_10_-RIB for detected agents by sampling region and year is presented in Figure 3A. Similar to ‘diversity,’ a significant increase in RIB was observed from freshwater to saltwater, but a distinct trend in the three regions of the marine environment (by year) was not evident. The interaction between sampling region and year was statistically significant (*P* = 0.026). In both Regions 1 and 2, RIB was significantly higher in 2013 than in 2012 (Figure 3B), but decreased between Regions 2 and 3 in 2013, but not 2012, resulting in closer RIB levels between the 2 years in Regions 3 and 4.

### Prevalences and Logistic Regression Models

**FIGURE 4:**
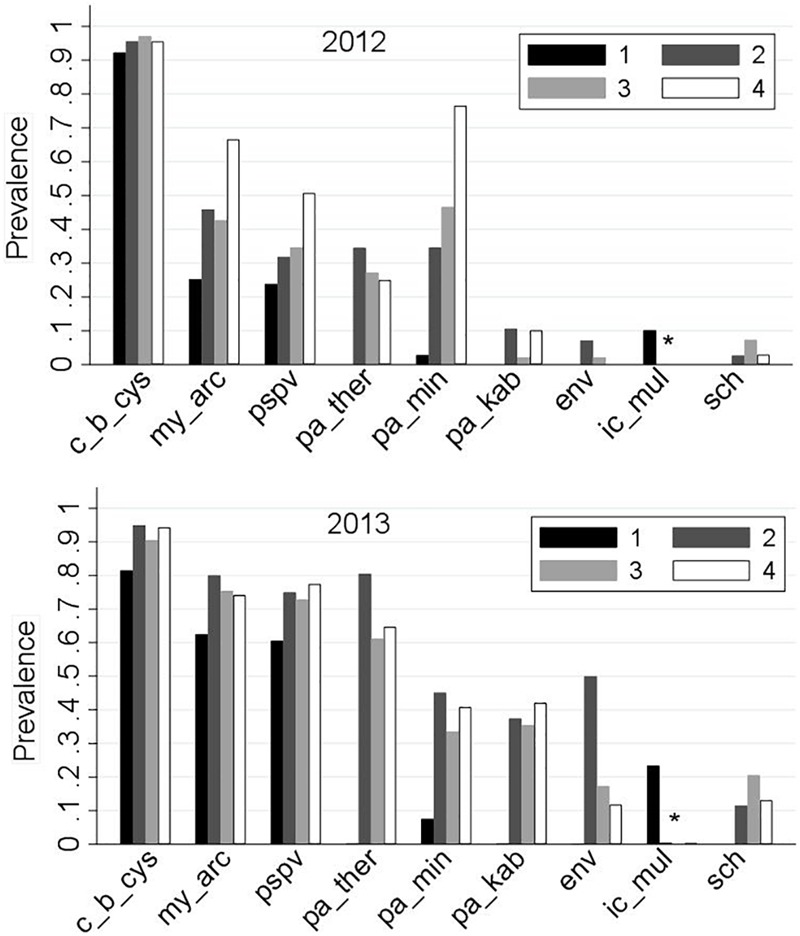
Distribution of the prevalences of the nine common infectious agents (as described in Table 1) for 2,006 Fraser River juvenile Sockeye salmon by sampling region and year. Sampling regions: (1) Freshwater, (2) Strait of Georgia, (3) Discovery Islands, and (4) Johnston Strait and north. ^∗^‘ic-mul’ is a freshwater pathogen.

Prevalences of the common infectious agents by sampling region and year are presented in Figure 4. Among the nine common agents, the following are known as marine agents: *Paraneuclospora theridion* (pa_ther; also known as *Desmozoon lepeoptherii*), *Parvicapsula kabatai* (pa_kab), erythrocytic necrosis virus (ENV), and gill chlamydia (sch). Consistent with their marine transmission, the prevalences of these agents in ‘Region 1’ were zero. *Ichthyophthirius multifiliis* (ic_mul) is a known freshwater pathogen, and it was principally observed in ‘Region 1,’ although it was detected in two saltwater samples in 2013 (Supplementary Table S3).

**FIGURE 5:**
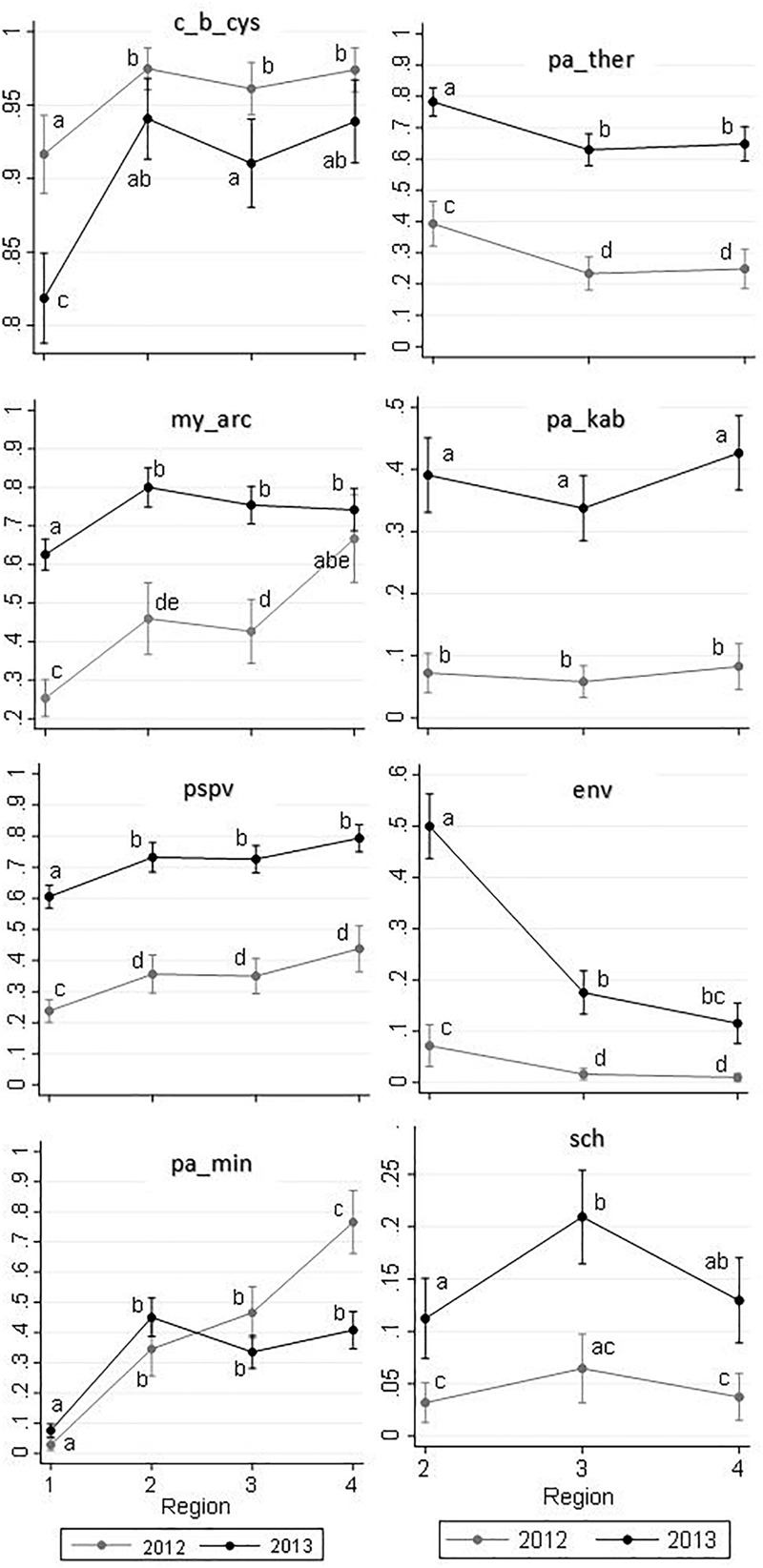
Predicted probabilities of infectious agent detections (*Y*-axes) in 2,006 Fraser River juvenile Sockeye salmon by sampling region (*X*-axes) and year, based on logistic regression models. Sampling regions: (1) Freshwater, (2) Strait of Georgia, (3) Discovery Islands, and (4) Johnston Strait and north. Small letters used on the plots represent statistically significant Bonferroni groups, where differences were observed when the points (i.e., unique combinations of region and year) do not have any letters in common (*P* < 0.05). If the points shared at least one letter with each other, they were not statistically different (*P* > 0.05). Saltwater agents are presented in the right column, which included regions 2–4.

Results of the logistic regression models for common agents are summarized in Figure 5. In this figure, relationships of the independent variables of interest (i.e., region, year, and their potential interaction) with the predicted probability of infection with any given agent are illustrated. The corresponding table of results is included in the Supplementary Table S4. The interaction terms between region and year was only significant for *M. arcticus* (*P* = 0.003) and *Parvicapsula minibicornis* (*P* < 0.001) (Figure 5). A brief description on the detection of each common agent is presented below.

The agent *C. B. cysticola* was detected in 90.2% of the samples. Its prevalence in freshwater was significantly higher in 2012 (92%) than in 2013 (82%). From freshwater to saltwater, the prevalence rose significantly, but the difference between the 2 years was not substantial afterwards (Figure 4). There were no statistically significant differences in prevalence among the three regions within the marine environment in each year (Figure 5 and Supplementary Table S4).

The agent *M. arcticus* had substantially higher prevalence in 2013 over all regions (70%), as compared to 2012 (37%). Based on Figures 4, 5, in 2013, the prevalence rose from 63% in freshwater to 80% in the Strait of Georgia, and did not change significantly afterwards. However, in 2012, the prevalence showed an overall increasing trend from 25% in freshwater to 66% in the northern region, where there was no statistically significant difference in prevalence between the two study years in the marine environment (Figure 5).

The overall prevalence of PSPV in 2013 (69%) was significantly higher than in 2012 (30%) in all four regions (Figures 4, 5). After a significant increase upon ocean entry, the prevalence did not show any significant changes along the migration route in the marine environment (Figure 5 and Supplementary Table S4).

The prevalence for the agent *P. minibicornis* showed a clear increasing trend from 3% in freshwater to 76% in the northern region in 2012. In 2013, the prevalence significantly increased from 8% in freshwater to 45% in the Strait of Georgia, but it did not change significantly afterwards (Figures 4, 5).

For all of the saltwater agents (i.e., *P. theridion, P. kabatai*, ENV, and gill chlamydia), overall prevalences were significantly higher in 2013 than in 2012 (Figure 5, right column). In general, differences in agents’ prevalences between freshwater and saltwater dominated over the regional differences; however, *P. theridion* and ENV showed a significant decrease in prevalence toward northern regions in both years (from Regions 2–3). In 2013, the reduction in prevalence of ENV was substantial, with a drop from 50 to 20% over the course of migration from the Strait of Georgia (Region 2) to Discovery Islands (Region 3). In contrast, fluctuations in the prevalence of *P. kabatai* and gill chlamydia along the migration route were not prominent (Figures 4, 5). The prevalence of *I. multifiliis* in freshwater was significantly higher (*P* < 0.001) in 2013 (23%) compared to 2012 (10%).

In all logistic regression models, the overall effects of sampling region and year on the prevalence of the individual agents were highly significant (*P* < 0.001 for all).

Because of the dominance and importance of Chilko stock in the overall trends observed in the prevalence of the common agents, corresponding prevalence distributions are also presented by the stock of origin (Chilko vs. all other stocks combined) in the Supplementary Figure S1 for discussion purposes. With the exception of a couple of freshwater agents specifically transmitted in the Chilko system (*M. arcticus* and PSPV, in 2012), these data showed that the resolution of spatial patterns in prevalences along the migration path was not substantively different when focusing on a single stock (i.e., Chilko), or multiple stocks combined.

### Loads and Linear Regression Models

**FIGURE 6:**
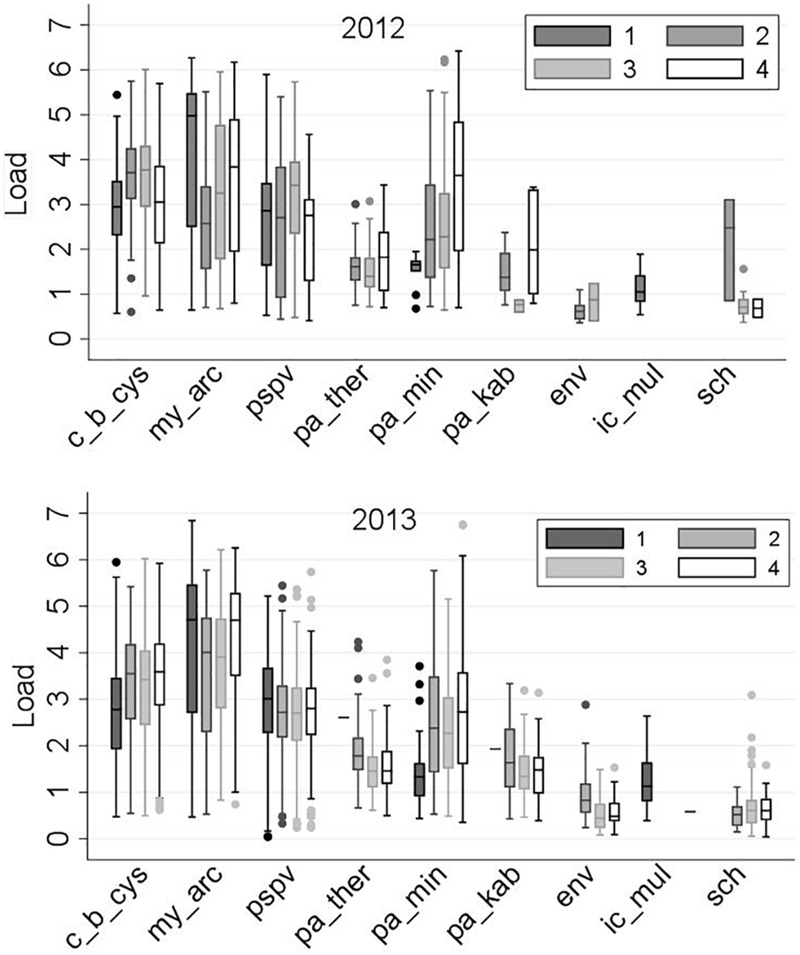
Distribution of the loads [log_10_ (copy number + 1)] of the nine common infectious agents (as described in Table 1) in 2,006 Fraser River juvenile Sockeye salmon by sampling region and year. Sampling regions: (1) Freshwater, (2) Strait of Georgia, (3) Discovery Islands, and (4) Johnstone Strait and north.

**FIGURE 7:**
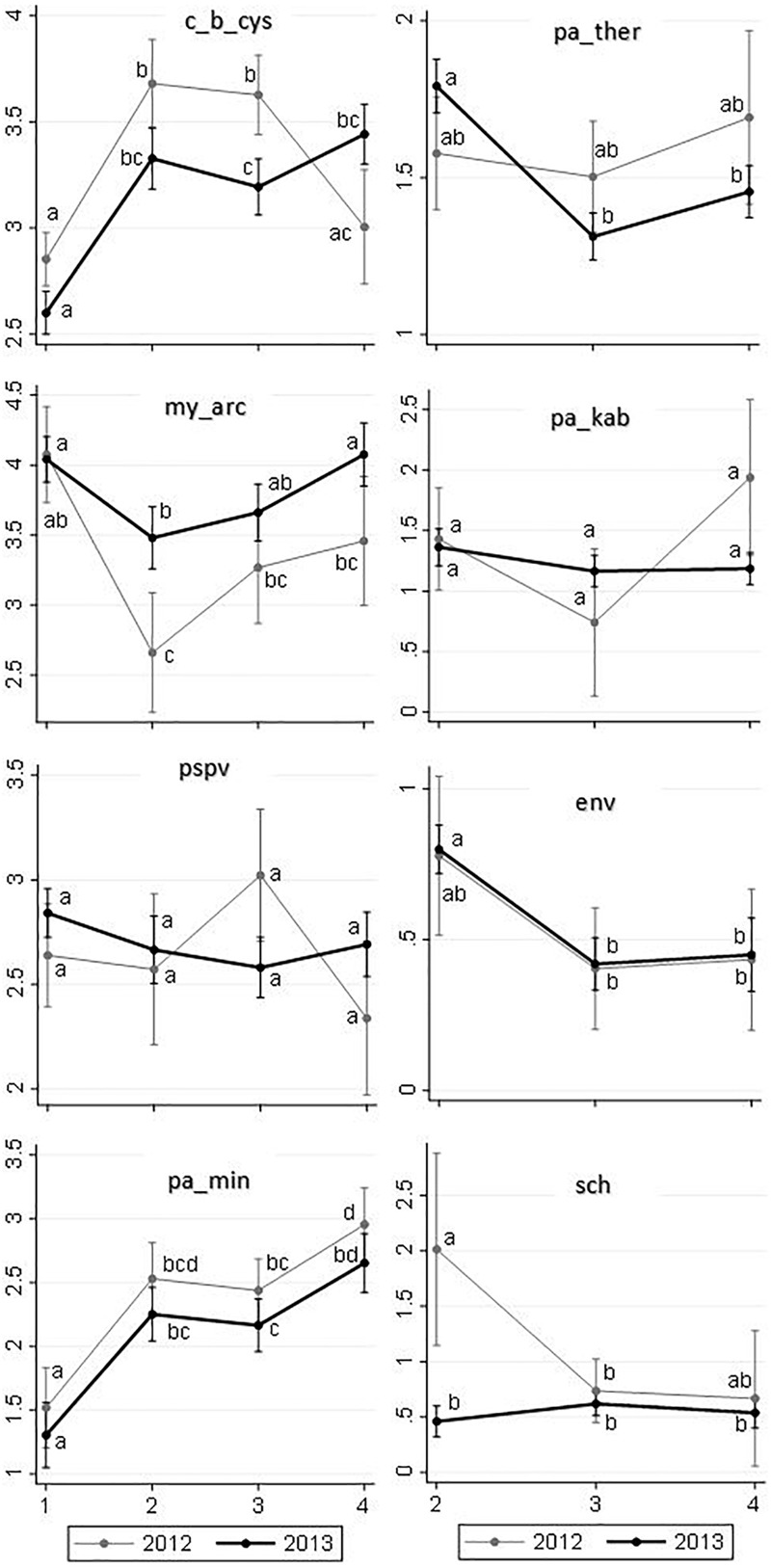
Predicted means of loads for the common infectious agents (*Y*-axes) detected in 2,006 out-migrating Fraser River Sockeye salmon, by study region (*X*-axes) and year, based on linear regression models. Sampling regions: (1) Freshwater, (2) Strait of Georgia, (3) Discovery Islands, and (4) Johnston Strait and north. Small letters used on the plots represent statistically significant Bonferroni groups, where differences were observed when the points (i.e., unique combinations of region and year) do not have any letters in common (*P* < 0.05). If the points shared at least one letter with each other, they were not statistically different (*P* > 0.05). Saltwater agents are presented in the right column, which included regions 2–4. A square root transformation was conducted on the loads of *P. theridion, P. minibicornis, P. kabatai*, ENV, and gill chlamydia in order to meet the normality and homoscedasticity assumptions in their respective linear regression models. The average loads for these agents have been back-transformed to the original scale. Presented 95% confidence interval for each point was calculated using delta method in Stata v15.1.

The frequency distributions of loads for the nine common infectious agents, by sampling region and year, are displayed in Figure 6. Overall, the loads did not show substantial differences between 2012 and 2013, with only a few exceptions, such as *M. arcticus* and gill chlamydia in Region 2. The corresponding linear regression models (indicating changes in the predicted mean of loads by sampling region and year) are illustrated in Figure 7. In addition, the corresponding table of results is included in the Supplementary Table S5.

Only two agents showed significant shifts in the load distribution among the sampling regions: *P. minibicornis*, whose load generally increased as salmon migrated northward in the marine environment, and ENV, which was first observed upon entry into saltwater (Region 2) and whose load decreased with migration toward Region 3 (Figure 7). However, it is notable that the number of observations (i.e., positively tested samples) in 2012 for the last five agents (especially, ENV and gill chlamydia) was scarce in some regions (Supplementary Table S3). For instance, for gill chlamydia in Regions 2 and 4, only three and two observations were available, respectively. As such, very wide confidence intervals for these agents were observed in 2012, and any interpretation on potential trends should be made cautiously.

## Discussion

Our study is the first comprehensive analysis of a broad range of infectious agents in juvenile Sockeye salmon from the Fraser River system, providing a baseline for the existence, prevalence, and burden of 45 known and putative pathogens, as well as one parasite of economic importance (*Kudoa thyrsites*) in Fraser River juvenile Sockeye salmon. As a part of a Cohen Commission technical report in 2011, the state of knowledge, at the time, on important infectious diseases and their potential impacts on survival of Fraser River Sockeye salmon were described (Kent, 2011). In that report, the following pathogens were introduced as potentially ‘high-risk’ agents: Infectious hematopoietic necrosis virus (IHNV), *Vibrio anguillarum, Aeromonas salmonicida, Renibacterium salmoninarum, Ichthyophthirius multifiliis*, and *Parvicapsula minibicornis*. In the present study, we demonstrated that only the last two agents (i.e., *I. multifiliis* and *P. minibicornis*) were prevalent in Fraser River Sockeye smolts in 2012 and 2013, whereas the rest of these agents (IHNV, *A. salmonicida, R. salmoninarum*, and *V. anguillarum*) were not detected. However, we note that IHNV, a well-documented endemic virus infecting Sockeye salmon (Kent, 2011) has been detected in Sockeye salmon sampled at the Chilko River fence (at the junction between the river and the lake) in previous studies, and has been associated with reduced migratory survival in freshwater (Jeffries et al., 2014; Miller et al., 2017). The Kent report (2011), commissioned by the Cohen inquiry, was prepared based on historic studies, government reports, and experts’ opinions. Thus, it did not include structured surveys of all important infectious agents (i.e., did not collect and test fish samples) or more recent reports since 2010. Importantly, many of the agents that we have observed in relatively high abundance were considered of low risk in the Kent report, merely because they had not previously been assessed in Sockeye salmon (e.g., erythrocytic necrosis virus = ENV); some agents were not considered at all, such as those associated with emerging diseases in Norway (e.g., *Paraneuclospora theridion*). Our study, therefore, begins to fill in the knowledge gaps specific to Sockeye salmon identified in the Kent report, and can possibly be considered in future prioritization for research and control of important infectious agents that may contribute to Fraser River Sockeye population declines. In addition to our general discussion, we present our findings on the most prevalent infectious agents (i.e., the top nine agents in Table 1) and what we know from the literature about their potential to cause diseases in the Supplementary Materials given the gap between what was presented in the Kent report (2011) and our study.

### Temporal Comparison of the Prevalent Agents

We showed a consistent and substantial difference in the prevalence, diversity, and infection burden for many infectious agents between the two study years, with nearly all of the estimates being significantly higher in 2013 than in 2012. This difference occurred consistently across all regions in freshwater and seawater, suggesting the influence of broad-scale annual drivers. One such driver could be the overall temperature during the out-migration period in the spring and summer. Interestingly, warmer ocean temperature anomalies (referred to as the ‘warm blob’) were first identified in the Northeast Pacific Ocean in 2013, with both marine and freshwater conditions warmer than usual in the spring of 2013 (Grant and Michielsens, 2017). While temperature can mediate a number of biological factors influencing salmon growth and survival, it has been shown that warmer weather (even within the normal range) has a potential positive impact on replication and abundance of many infectious agents (reviewed in ‘Miller et al., 2014’). In addition to the impacts of temperature on pathogen growth and host immunity, rapid changes in temperature may act as a stressor that can alter host resistance (Purcell et al., 2016). One exception to the overall year effect was the prevalence of *Candidatus Brachiomonas cysticola*, which was higher in freshwater in 2012 compared to 2013. This mainly resulted from higher prevalence of *C. B. cysticola* in the rearing lakes other than Chiko in 2012, compared to 2013.

For out-migrating smolts, 2013 corresponded to the 2015 return year, general productivity (Ln (returns/spawner)) was below 0, which is very similar to what was observed in the 2009 crisis with record-low annual returns (Grant and Michielsens, 2017). This low productivity was associated with poor marine survival of fish, with survival in the first 3–6 months being the key determinant of year-class strength in most years (Beamish and Mahnken, 2001). In contrast, the 2012 out-migrating smolts, which returned to spawn in 2014, experienced an average productivity, hovering just above 1.2. This occurred during a dominant cycle year that resulted in 21 million fish returning to spawn (Grant and Michielsens, 2017). Although 2 years of data is not adequate to find a general temporal trend in prevalence and burden of infectious agents in out-migrating wild Pacific salmon, the finding that the majority of infectious agents were more prevalent in smolts from one of the poorest years of productivity on record is intriguing. Moreover, our data suggest that in 2013, Sockeye salmon already carried higher overall infection burden, even prior to entering the ocean. Upon ocean entry, there continued to be higher prevalences of multiple saltwater agents in 2013 compared to 2012, resulting in a further increase in overall infection burden in the fish sampled from the Strait of Georgia (Region 2) in 2013. While high water temperatures may have contributed to the higher prevalence (and perhaps transmission) of infectious agents in the ocean in 2013, it is also possible that fish compromised by a high infection burden in 2013 experienced enhanced susceptibility to infection. The sharp reduction in infection burden noted between the Strait of Georgia (Region 2) and the Discovery Islands (Region 3) in 2013 (but not in 2012) suggests evidence for a potential link between infection burden and survival, as we hypothesize that there would have been limited chance for these fish to recover from an array of new infections during such a short period of time (estimated at 43–54 days on average by Preikshot et al., 2012), especially in this critical period when they are also adapting to a new salinity environment.

The largest reduction of infection burden in fish between Regions 2 and 3 in 2013 were associated with ENV and *P. theridion*, both transmitted upon arrival to the marine environment; however, only ENV had a dissimilar pattern between the study years. In 2013, ENV was observed at a prevalence of 50% in Region 2, dropping to <20% in Region 3; and retained a prevalence of <10% across Regions 2 and 3 in 2012. ENV is a saltwater iridovirus that causes viral erythrocytic necrosis (Emmenegger et al., 2014) in multiple species of salmon with the most notable pathological effects in herring (Evelyn and Traxler, 1978; Eaton, 1990; Hershberger et al., 2006). To our knowledge, this virus has not specifically been studied in Sockeye salmon. ENV replicates in ‘viral factories’ or inclusion bodies within erythrocytes (EIBS), and can cause severe anemia and reduction in stamina. The virus can also predispose fish to other infections, and/or increase the impact of other stressors (e.g., low oxygen) and predation, at times leading to population-level impacts in susceptible species (Winton and Hershberger, 2014). Given what is already known about high mortality rates during the critical early period in the marine environment, and the importance of gill health in transitioning between salinity environments, we hypothesize that in 2013 the high infection prevalence with ENV could have contributed to early marine mortality. Our study suggests that ENV warrants a closer look in terms of potential to cause disease in Sockeye salmon. *P. theridion* (also known as *Desmozoon lepeoptherii*) is a microsporidian parasite carried and possibly transmitted by sea lice in saltwater. This parasite was discovered in farmed Atlantic salmon in western Norway in 2008, and is considered a primary agent in cases with high mortality linked to proliferative gill disease in Norway (Nylund et al., 2010, 2011). Future studies will be required to elucidate the potential pathogenic effects of *P. theridion* in juvenile Sockeye salmon or in Pacific salmon in general.

### Spatial Distribution of the Prevalent Agents

The prevalence and burden of the common infectious agents found in both freshwater and marine environments showed a significant rise upon ocean entry in each study year. This finding is consistent with studies conducted on other Pacific salmon species (Thakur et al., 2018; Tucker et al., 2018). For saltwater-specific agents, the first exposure often occurs in the mouth of Fraser River and the Strait of Georgia. Within the marine environment (Regions 2–4), the prevalence and burden of common agents did not change substantially over both study years, with a few exceptions, such as *P. minibicornis* in 2012, *P. theridion* and ENV (as discussed above).

Two of the most prevalent agents known to be transmitted in the freshwater environment, *P. minibicornis* and *M. arcticus* showed increasing prevalence upon entry to the marine environment, a result that was not expected but has recently been observed in juvenile Chinook salmon (Tucker et al., 2018). High estuarine abundance of the alternate host for *P. minibicornis, Manayunkia speciosa*, likely contributed to the sharp increase in the prevalence and load between Regions 1 (freshwater) and 2 (saltwater) (Kent, 2011; Mahony et al., 2017). In 2012 alone, both agents experienced a sharp rise in prevalence from Region 3 to 4. We suspect that new exposures to freshwater pathogens could have occurred in this region as a result of the melting of heavy snowpack and unusually high rainfalls throughout BC in spring–summer 2012 (Environment and Climate Change Canada, 2018).

Among the most prevalent infectious agents in our study, those with strongest demonstrated pathogenic potential in salmon include ENV (discussed above), *P. minibicornis*, and *I. multifiliis*. While *M. arcticus* has been associated with abnormal swimming behavior in naturally infected smolts (Moles and Heifetz, 1998), it appears to cause negligible direct pathological effects (Urawa et al., 2011). *P. minibicornis* typically causes lesions in kidney tissue, but it is also associated with branchitis, osmoregulatory dysfunction, and pre-mature mortality in returning adult Sockeye in the Fraser River (Bradford et al., 2010). Given the link with parasite-associated mortality in freshwater, Kent (2011) categorized *P. minibicornis* as a high-risk pathogen to Sockeye salmon. Juvenile Sockeye salmon infection with *P. minibicornis* was also associated with increased risk of predation by Rhinoceros Auklets in the marine environment (Miller et al., 2014), although pathogenic effects of this parasite have not been specifically studied in this life history stage. *I. multifiliis* is a freshwater protozoan parasite which causes white spot disease (WSD) or ‘Ich’ in fish, and can cause high levels of mortality if it is not controlled (Durborow et al., 1998). Outbreaks of *I. multifiliis* were reported in adult pre-spawning and spawning Sockeye salmon during 1994 and 1995 in the Skeena River watershed, northern BC, leading to substantial mortality (Traxler et al., 1998). Severity of the disease and mortality can rise with increased water temperature and low water flow. *I. multifiliis* was considered as a high-risk pathogen to Sockeye salmon in the Kent report (2011). However, given very low prevalence (to zero) in saltwater, its impact is likely restricted mostly to freshwater.

Two of the prevalent agents in our study that were not included in the Kent report (Kent, 2011) and have not yet been associated with any specific disease in Sockeye salmon were PSPV and *Parvicapsula kabatai.* PSPV is a DNA virus that was initially identified by our team via high throughput sequencing of Sockeye salmon tissues (Miller et al., 2011). Infection with this virus has been reported in both freshwater and saltwater (Miller et al., 2014). It appears that Sockeye salmon may be the principle host for PSPV (Miller et al., 2017). *P. kabatai* was first described in kidney tissue from Pink salmon from the Quinsam River, BC (Jones et al., 2006). This saltwater parasite has been associated with increased likelihood of predation of juvenile Sockeye salmon by Rhinoceros Auklets (Miller et al., 2014). There is no information as of yet on the potential pathogenic effects of these agents in Sockeye salmon.

### Low-Prevalent Infectious Agents

Additional agents were detected at a prevalence of <3% in our samples, such as *Flavobacterium psychrophilum, Parvicapsula pseudobranchiocola, Ichthyophonus hoferi, Ceratonova shasta*, with some individuals carrying appreciable loads of the latter three. *F. psychrophilum* was mostly observed in freshwater (Region 1), but the latter three were more common in the marine environment. All of these agents can potentially cause diseases, with *C. shasta* being reportable to the Canadian Food Inspection Agency. We were not able to evaluate the spatiotemporal shifts for these agents due to low number of positive samples, but they were included in calculations and analyses of the overall diversity and infection burden. Some of the most virulent viral agents that have substantially affected farmed salmonids across the world, namely, infectious salmon anemia virus (ISAV), infectious pancreatic necrosis virus (IPNV), salmonid alphavirus (SAV), and *Oncorhynchus masou* herpesvirus (OMV), were not detected in any of our samples.

The marine sampling locations were divided into three regions (Regions 2–4) to capture the potential interaction between out-migrating Sockeye salmon and salmon farms, by contrasting the agents’ distributions before (Region 2), during (Region 3), and post initial (Region 4) contact with farming regions. However, we must be cognisant of the fact that our sampling design may not be sensitive enough to detect all transmission events (if any) given the rapid migration (approximately 2 weeks) of juvenile Fraser River Sockeye through the passageway harboring the highest density of farms in BC (Region 3: Discovery Islands through Broughton Archipelago). Moreover, assuming a similar speed of migration, fish would have reached the northern end of Haida Gwaii (our most northerly sampling location) within only 3–4 weeks post farming regions. Hence, the potential transmission of agents that replicate slowly and take time to reach detectable copy numbers might have been missed. On the flip side, limited detection of new agents after potential contacts with farms could also derive from a limited role of farms in the transmission of these infectious agents. In general, the spatial distribution of infectious agents across the farming regions may not indicate their potential sources. For instance, some agents may originate from other sources/reservoirs than farms (e.g., other species) and spread through both farmed and wild salmon in the region.

Our study clearly shows that the vast majority of infectious agents detected in juvenile salmon in the spring and summer were naturally occurring components of freshwater and marine ecosystems, with five of the nine most common agents emanating from freshwater, and the remaining detected upon entry into the ocean. However, there were seven infectious agents with rare detections around and after the farming regions (i.e., in Regions 3 and 4). These included Piscine orthoreovirus (PRV), *Piscirickettsia salmonis, Moritella viscosa, Tenacibaculum maritimum, Facilispora margolisi, K. thyrsites*, and *Nanophyetus salmincola.* Of these, *N. salmincola* is not a likely candidate for being transmitted from farmed to wild salmon because this is a freshwater-transmitted trematode, observed in a single fish in 2012. This latter detection provides further evidence that there may have been some exposure to freshwater influence during the passage between Regions 3 and 4 in 2012. Transmission of some these agents may have been influenced by farm activities. Prevalence of PRV on salmon farms in BC is generally >60% (Marty et al., 2015; Laurin et al., 2019). In our study, PRV was detected in a single fish from Hecate Strait, Haida Gwaii, but with an appreciable load (3.14 or >10^3^ copy numbers). Given that the latent period for PRV infection is estimated at a minimum of 2–3 weeks (Polinski et al., 2016), it is likely that we would have missed more infected fish (if any) due to our sampling timeline, which did not extend beyond this window. On the other hand, PRV has been detected from other wild Pacific salmon species in regions without salmon farms, such as Alaska (Purcell et al., 2017). *P. salmonis* has been detected in >20% of farmed fish (Laurin et al., 2019), and was detected in a single Sockeye salmon sampled from Region 4. This bacterium can cause acute disease and is generally detected during disease outbreaks (Rozas and Enríquez, 2014). While not a known salmon pathogen, *F. margolisi* is a parasite observed commonly in sea lice and can be transmitted to salmon (Jones et al., 2012). This parasite has been observed in farmed and wild fish in our program (Thakur et al., 2018; Tucker et al., 2018; Laurin et al., 2019). High sea lice densities around the farms could theoretically contribute to increased exposure of migratory salmon to this parasite. *K. thyrsites* is not a pathogen, but rather a myxozoan parasite that causes post-mortem myoliquefaction of muscle tissues, and hence affects the marketability of fish. It has been reported around the Discovery Islands (Laurin et al., 2019) and may have been concentrated in this region even before the salmon industry became established (unpublished data). These data could be considerably strengthened with additional years of study and sequence-level data to determine if detected strains from migratory wild salmon are the same as those existing around farming regions. Interestingly, we did not detect two agents that are somewhat common in farmed salmon, namely *R. salmoninarum* (>30%) and *V. anguillarum* (>10%) (Laurin et al., 2019).

### Importance of the Chilko Stock

The Chilko stock typically contributes substantial proportions to the total returns in most years, and is the indicator stock for Fraser River Sockeye salmon. As a result, Chilko is the only Sockeye stock for which total survival can consistently be divided into freshwater and marine survival over a long period of time (Grant and Michielsens, 2017). In our study, Chilko was the only stock sufficiently abundant across the 2 years of sampling to allow for the evaluation of spatiotemporal changes in agents’ prevalences within a stock (only enough for the top seven agents in Table 1). We contrasted the agents’ prevalences between the Chilko stock and all other stocks combined, and showed that while there were differences in the prevalence of common infectious agents transmitted in the freshwater environment, there were no substantive differences in marine-transmitted agents. We hypothesize that the variation in the freshwater profiles likely derives from the distinct distribution and diversity of infectious agents within different nursery lakes and rivers across the Fraser River watershed (Kent, 2011). However, because freshwater samples in our study were taken from both natal rearing areas and the lower Fraser River (where stocks would have come together in a common environment), we expect that the stock-specific divergence could have been even stronger if the contrasts had focused strictly on the natal rearing areas. The lack of differentiation in the prevalences between Chilko and other stocks in the ocean is consistent with another study undertaken by our team, contrasting hatchery and wild Chinook salmon profiles (Thakur et al., 2018). That study suggested that the sampling environment of the fish contributed more to variation in marine-transmitted agent prevalence and burden than the freshwater origin of the fish (Thakur et al., 2018). However, another study in juvenile Chinook salmon that contrasted the agent distributions across life-history types (stream- and ocean-type stocks) found that the differences persisted in the marine environment (Tucker et al., 2018). In the latter study, we expected that differences in timing of ocean entry, size of fish, and habitat utilization between yearling and sub-yearling Chinook salmon stocks contributed to the persistence of variation in infectious agent profiles. The study herein did not include the only Fraser River Sockeye salmon stock with an ocean-type life history (i.e., Harrison), which would provide a validation of our observations in Chinook salmon.

Our future studies will attempt to gain further insight into the factors contributing to variation in infectious agent profiles within habitats [e.g., stock, life-history, origin (hatchery/wild)], and between habitats, seasons, and years of high and low productivity. In addition, the potential interaction between farmed and wild salmon with respect to the common pathogens will be investigated.

## Conclusion

In the first large-scale survey of infectious agents in juvenile Sockeye salmon in BC, among the 26 detected agents, some were not previously associated with Sockeye salmon. We showed that the prevalences of the most common pathogens were significantly higher for a year-class (2013 out-migrants) that experienced low productivity than one with average productivity (2012 out-migrants). This also led to a greater pathogen diversity and overall infection burden in 2013. In 2013, a sharp decline in the prevalence and load of one pathogen, ENV, within the 1st month of ocean residence was noteworthy – this pattern of detection is consistent with pathogen-associated mortalities, but additional research is required to establish the potential pathogenicity of this virus in Sockeye salmon. A second agent, *P. theridion*, declined in prevalence over the 1st month of ocean residence in both study years, similar to observations in Chinook salmon (Tucker et al., 2018). Finally, we showed that while all commonly observed agents were natural components of freshwater and marine ecosystems, there were seven infectious agents that were first detected around and after salmon farming regions, albeit at minimal levels. Future studies over long periods of time (e.g., 10 years) are needed to establish the consistency of our findings, notably whether agents’ profiles are correlated with annual marine survival.

## Data Availability Statement

The raw data supporting the results of this manuscript will be made available by the authors, upon receiving a reasonable request from researchers in the field.

## Ethics Statement

The animal care and use protocol for this work was approved by the DFO Pacific Region Animal Care Committee (Animal Use Protocol Number: 13-008).

## Author Contributions

ON analyzed the data and led the writing of the manuscript. RV and KT advised on data analyses and provided technical support in the preparation of the manuscript. TM, KK, AT, and TB conducted the laboratory analyses and assisted in the preparation of the manuscript. EL and ST provided technical advice in writing the manuscript. KM led the project and provided technical support in the preparation of the manuscript.

## Conflict of Interest Statement

The authors declare that the research was conducted in the absence of any commercial or financial relationships that could be construed as a potential conflict of interest. The reviewer JJA declared a shared affiliation, with no collaboration, with one of the authors KM to the handling Editor at the time of review.

## Footnotes

1 http://www.qgis.org
