# Supplementary material for: Detection and Assessment of the Distribution of Infectious Agents in Juvenile Fraser River Sockeye Salmon, Canada, in 2012 and 2013
Source: Front Microbiol. 2018 Dec 21;9:3221. doi: 10.3389/fmicb.2018.03221 (PMC6309813; doi:10.3389/fmicb.2018.03221)

Supplementary materials:

Supplement discussion

Details on the nine common infectious agents detected in out-migrating Fraser River Sockeye salmon collected in 2012 and 2013, and what is known about their pathogenic potential

Candidatus Brachiomonas cysticola (c_b_cys) was first identified in BC salmon by Miller et al. (2014), and has since been observed in most Pacific salmon species with high prevalence in smolts and adults (Miller et al. 2017; Tucker et al. 2018; Thakur et al. 2018). *C. B. cysticola* was the most prevalent infectious agent in both freshwater and seawater in our study and was the only agent more prevalent in 2012 than 2013, largely owing to a higher prevalence in non-Chilko stocks. This bacterium is also prevalent in Norway and Ireland and is associated with proliferative gill inflammation in net pens, but its role in the development of the disease has not yet been established (Toenshoff et al. 2012, Mitchell et al. 2013). In a similar study conducted by our team on fish obtained from the regulatory Fish Health Audit Program of DFO in BC between 2011 and 2013, 897 recently-dead and dying fish from 15-30 randomly selected Atlantic and Pacific salmon farms were examined using the same qPCR platform as described herein (Laurin et al. in review). On the studied farms, the prevalence of *C. B. cysticola* in farmed Pacific salmon (principally Chinook salmon) was approximately 90% (also the most prevalent agent), and only 12% in Atlantic salmon, potentially suggesting a higher infection susceptibility of Pacific compared to Atlantic salmon.

Myxobolus arcticus (my_arc) is a myxozoan parasite infecting the anterior spinal cord or hindbrain of Sockeye salmon. While *M. arcticus* has been associated with abnormal swimming behavior in naturally infected smolts (Moles & Heifetz 1998), it appears to cause negligible direct pathological effects (Urawa et al. 2011). This parasite is endemic to BC and is known to infect juvenile salmon in freshwater, with prevalence varying substantially between years (Mahony et al. 2017), consistent with our findings, where we detected a much higher prevalence in 2013 than 2012. Its prevalence has been associated with the abundance of its annelid intermediate host (*Stylodrilus heringianus*) in major Fraser River stocks, such as Chilko (Kent et al. 1993). Indeed, we observed higher prevalence of this parasite in fish from the Chilko stock compared to all other Fraser River stocks combined. The observed increase in the overall prevalence of infection from freshwater to saltwater could be explained by continued exposure to the parasite in the lower river, although this pattern was opposite for the Chilko stock alone. The fact that overall load of *M. arcticus* decreased from Region 1 to 2 (freshwater to saltwater) is consistent with a larger proportion of very recently infected fish in Region 2. Interestingly, while there was approximately 30% difference in the prevalence of *M. arcticus* between 2012 and 2013 in Regions 1-3, a sharp rise in the prevalence from Region 3 to 4 in 2012 closed the large prevalence gap between the two years. We suspect that new exposures to freshwater pathogens (including *M. arcticus*) occurred in this region in 2012 as a result of heavy snowpack and unusually high rainfalls throughout BC in spring-summer (https://ec.gc.ca/meteo-weather/default.asp?lang=En&n=47EF1277-1).

Pacific salmon parvovirus (PSPV) is a DNA virus that was initially identified by our team via high throughput sequencing of Sockeye salmon tissues that contained a mortality-related genomic signature (MRS; defined in Miller et al. 2011). Infection with this virus has been

reported in both freshwater and saltwater (Miller et al. 2014). Herein, PSPV was highly prevalent, especially in 2013 (>60%). Thakur et al. (unpublished data) detected the virus as early as 1985 in archived adult Sockeye samples, also at high levels of prevalence (overall, 49%), indicating that the virus has likely been endemic to BC for an extensive period of time. In contrast, PSPV was not detected from juvenile Fraser River Chinook salmon (Miller et al. 2017, Tucker et al. 2018), nor was it observed in farmed Altantic or Chinook salmon (Laurin et al. in review). Based on these findings, and unpublished data from other species, it appears that Sockeye salmon may be the principle host for this virus. PSPV has not yet been associated with any specific disease in Sockeye salmon (Miller et al. 2017).

Parvicapsula minibicornis (pa_min) is an endemic freshwater-transmitted myxozoan parasite which has been observed across multiple Pacific salmon species (True et al. 2009). In our study, the parasite was observed at the similar levels of prevalence in both years (<8% in freshwater and >30% in saltwater), but as with *M. arcticus*, its prevalence significantly increased from freshwater to saltwater. In 2012 alone, prevalence sharply rose in Region 4, consistent with additional exposure to freshwater (in a year of heavy rainfall) during migration through the narrow corridor from Discovery Islands through Johnstone Strait. High estuarine abundance of the alternate host for *P. minibicornis*, *Manayunkia speciosa*, likely contributed to the sharp increase in the prevalence and load between Regions 1 and 2 (Kent 2011, Mahony et al. 2017). An increase in the prevalence and load for *P. minibicornis* over migration in the marine environment has been observed previously, e.g. Mohony et al. (2017) for Sockeye and Tucker et al (In press) for Chinook salmon smolts. In Fraser River adult salmon, *P. minibicornis* prevalence and load also increased during freshwater migration towards spawning grounds (Hilaire et al. 2002, Teffer et al. 2017). *P. minibicornis* typically causes lesions in kidney tissue, but is also associated with branchitis, osmoregulatory dysfunction, and pre-mature mortality in returning Sockeye in the Fraser River (Bradford et al. 2010). Given the link with parasite-associated mortality in freshwater, Kent (2011) categorized *P. minibicornis* as a high-risk pathogen to Sockeye salmon. While its pathogenicity has not directly been studied in saltwater smolts, a sharp reduction in the prevalence and load was observed between seasons in juvenile Chinook salmon, and considered consistent with parasite-associated mortality in the marine environment (Tucker et al, In press); histopathology findings confirmed that lesions were present preceding the decline in prevalence and load (Emiliano Di Cicco, unpublished data). Juvenile Sockeye salmon infection with *P. minibicornis* was also associated with increased risk of predation by *Rhinoceros Auklets* in the marine environment (Miller et al. 2014).

Paraneuclospora theridion (pa_ther; also known as *Desmozoon lepeoptherii*) is a microsporidian parasite carried and possibly transmitted by sea lice in saltwater. This parasite was discovered in farmed Atlantic salmon in western Norway in 2008, and is considered a primary agent in cases with high mortality linked to proliferative gill disease in western Norway (Nylund et al. 2010, 2011). In our study, *P. theridion* was first detected in saltwater (Region 2), with >35% higher prevalence in 2013 than 2012. In both years, prevalence decreased from Region 2 to Region 3 despite our expectation of higher densities of sea lice around the farms. Notably, in 2013 (but not 2012), the load of *P. theridion* also sharply diminished in Region 3 and remained low thereafter, consistent with parasite-associated mortality pattern. A similar truncation in load was observed between seasons (fall-winter) in juvenile Chinook salmon (Tucker et al. 2018). Laurin et al. (in review) found that the prevalence of infection was also high in farmed dead-and-dying salmon, between 60% (Pacific salmon farms) and 90% (Atlantic salmon farms). Nylund et al. (2011) hypothesized that *P. theridion* predisposes the host to other infectious agents. In the present

study, 619 cases of the 622 fish with *P. theridion* had at least another agent (co-detections), and the average 'diversity' for *P. theridion*-positive samples (4.7) was significantly higher than that for *P. theridion*-negative samples (2.5) (results not shown). Future studies will be required to elucidate the exact pathogenesis of *P. theridion* in juvenile Sockeye salmon or in Pacific salmon in general.

Parvicapsula kabatai (pa_kab) was first described in kidney tissue from Pink salmon from the Quinsam River, BC (Jones et al. 2006). In our study, *P. kabatai* was first detected in saltwater (Region 2) and maintained at 30% higher prevalence in 2013 than 2012. This saltwater parasite has been associated with increased likelihood of predation of juvenile Sockeye salmon by *Rhinoceros Auklets* (Miller et al. 2014). Prevalence of *P. kabatai* in juvenile Sockeye salmon is higher than the prevalences observed in juvenile Chinook salmon and aquaculture audit samples of Pacific and Atlantic salmon, both < 5% (Tucker et al. 2018); Laurin et al. in review). There is no information as of yet on the pathogenic effects of this parasite.

Erythrocytic necrosis virus (ENV) is a saltwater iridovirus that causes viral erythrocytic necrosis (Emmenegger et al. 2014) in multiple species of salmon with the most notable pathologic effects in herring (Evelyn & Traxler 1978, Eaton 1990, Hershberger et al. 2006). The virus, which replicates in 'viral factories' or inclusion bodies within erythrocytes (EIBS), can cause severe anemia and reduction in stamina. ENV can also predispose fish to other infections, and/or increase the impact of other stressors (e.g. low oxygen) and predation, leading to population-level impacts in susceptible species (Winton & Hershberger 2014). In our study, ENV was first detected in Region 2, with a prevalence > 50% in 2013 and < 10% in 2012. From Region 2 to 3, a precipitous decline in prevalence and a slight reduction in load were observed. It takes more than a month for Sockeye salmon to travel from the mouth of Fraser River to Discovery Islands (Preikshot et al. 2012); therefore, it is unlikely that the fish would have recovered from this virus in that period of time. We hypothesize that the observed decrease was more likely due to mortality. Similar prevalence levels were reported for juvenile Chinook salmon (Tucker et al. 2018) and dead-and-dying farmed Pacific salmon (Laurin et al. in review), but lower prevalence was observed in dead-and-dying farmed Atlantic salmon (< 15%) (Laurin et al. in review). In the Kent report (2011), ENV was considered a high-risk pathogen because it could cause severe infection and potentially significant disease at the population level. Given the results of our study, we recommend further attention be paid to this virus and its potential for pathogenic effects on wild salmon.

Ichthyophthirius multifiliis (ic_mul) is a freshwater protozoan parasite which causes white spot disease (WSD) or 'Ich' in fish, and can cause high levels of mortality if it is not controlled (Durborow et al. 1998). We observed this parasite in freshwater at an overall prevalence of < 10%. *I. multifiliis* has been detected at low prevalence (< 5%) from dead-and-dying farmed salmon (Laurin et al. in review) and wild juvenile Chinook (Tucker et al. 2018). Outbreaks of *I. multifiliis* were reported in adult pre-spawning and spawning Sockeye salmon during 1994 and 1995 in the Skeena River watershed, northern BC, leading to substantial mortality (Traxler et al. 1998). Severity of the disease and mortality can rise with increased water temperature and low water flow. *I. multifiliis* was considered as a high-risk pathogen to Sockeye salmon in the Kent report (2011). However, given low prevalence in saltwater, its impact is likely restricted mostly to freshwater.

Salmon gill chlamydia (sch) was originally detected in farmed Atlantic salmon suffering from proliferative gill disease in Europe (Duesund et al. 2010). Consistent with our findings, low

prevalence of this infection (< 10%) has also been reported in wild juvenile Chinook salmon (Tucker et al. 2018) and in dead-and-dying farmed salmon in BC (Laurin et al. in review). There is a clear knowledge gap around the pathogenesis and exact role of this agent in proliferative gill disease, which demands additional studies.

Note: references have been included in the main manuscript.

Table S1. Distribution of the fork-length (median and 5-95 percentiles in ‘mm’) of 1,717 Fraser River juvenile Sockeye salmon in this study by region and year [a].

Year	Region	N	Median	5%	95%
2012	1. Freshwater	323	73	61	97
	2. Strait of Georgia	108	98	15	141
	3. Discovery Islands	139	96	76	124
	4. Johnstone Strait and north	69	130	91	211
2013	1. Freshwater	573	84	51	108
	2. Strait of Georgia	156	105	92	137
	3. Discovery Islands	205	103	89	140
	4. Johnstone Strait and north	144	112	101	138

[a] length data were available for 1,717 of 2,006 study fish.

Table S2. Frequency distribution of the stocks of origin for 2,006 Fraser River juvenile Sockeye salmon in the study.

Stock of origin	Frequency	%
1. Chilko	697	34.75
2. L Adams	182	9.07
3. L Shuswap	152	7.58
4. Stellako	91	4.54
5. Seymour	83	4.14
6. Pitt	78	3.89
7. Chilko south	73	3.64
8. Thompson N	71	3.54
9. Gates Cr	63	3.14
10. U Horsefly	63	3.14
11. Mitchell	62	3.09
12. Cultus Lake	61	3.04
13. MiddleShuswap	53	2.64
14. Scotch	46	2.29
15. Chilliw lake	34	1.69
16. Portage Cr	34	1.69
17. Birkenhead	27	1.35
18. DollyVarden Cr	21	1.05
19. Raft	18	0.90
20. Nahatlatch R	16	0.80
21. Wasko-Roaring	16	0.80
22. Bowron	9	0.45
23. Horsefly	8	0.40
24. Quesnel Mitche	8	0.40
25. Quesnel Horsef	7	0.35
26. Eagle L	5	0.25
27. L Horsefly	4	0.20
28. Chilko North	3	0.15
29. Little Shuswap	3	0.15
30. Upper Barrier	3	0.15
31. Weaver	3	0.15
32. Big Silver	2	0.10
33. M Horsefly	2	0.10
34. Nadina	2	0.10
35. Blue Lead Cr	1	0.05
36. Cayenne	1	0.05
37. Dust-Sinta	1	0.05
38. FiveMile	1	0.05
39. Middle R	1	0.05
40. Sandpoint	1	0.05
Total	2,006	100

Table S3. Prevalence, total number of samples (with conclusive results), and number of test-positive samples for the nine common infectious agents from 2,006 Fraser River juvenile Sockeye salmon, by sampling region and year (top row: prevalence; bottom row: number of positives / total). Sampling regions: 1) Freshwater, 2) Strait of Georgia, 3) Discovery Islands, and 4) Johnstone Strait and north. For infectious agent's complete name, refer to Table 1.

Year	Region	c_b_cys	my_arc	pspv	pa_ther	pa_min	pa_kab	env	ic_mul	sch
	1	0.923	0.253	0.238	0	0.028	0	0	0.101	0
		298/323	80/316	76/319	0/323	9/318	0/323	0/323	31/307	0/323
	2	0.956	0.459	0.318	0.345	0.346	0.106	0.071	0	0.027
2012		109/114	51/111	35/110	38/110	37/107	12/113	8/112	0/112	3/113
	3	0.971	0.426	0.346	0.272	0.466	0.022	0.022	0	0.073
		135/139	58/136	46/133	37/136	62/133	3/139	3/139	0/139	10/137
	4	0.956	0.667	0.507	0.250	0.766	0.101	0	0	0.029
		65/68	44/66	34/67	17/68	49/64	7/69	0/68	0/68	2/68
	1	0.815	0.626	0.606	0.004	0.076	0.003	0.002	0.234	0
		463/568	351/561	341/563	2/571	43/568	2/573	1/573	123/526	0/573
	2	0.950	0.800	0.750	0.805	0.451	0.374	0.500	0.005	0.115
2013		226/238	188/235	177/236	190/236	105/233	88/235	118/236	1/215	27/235
	3	0.906	0.754	0.728	0.612	0.336	0.355	0.173	0	0.205
		269/297	224/297	217/298	178/291	98/292	104/293	51/295	0/292	61/297
	4	0.944	0.742	0.775	0.648	0.408	0.421	0.118	0.004	0.132
		235/249	184/248	193/249	160/247	100/245	104/247	29/245	1/229	32/243

Table S4. Results of logistic regression models, evaluating the associations between sampling region and year, and the prevalence of each common infectious agent (dichotomous outcome). Sampling regions: 1) Freshwater, 2) Strait of Georgia, 3) Discovery Islands, and 4) Johnston Strait and north. For infectious agent's complete name, refer to Table 1.

Agent	Variable	Level	Odds ratio	95% CI L	95% CI U
c_b_cys	Region	1	Ref.[a]		
		2	3.51	2.08	5.93
		3	2.25	1.50	3.39
		4	3.40	2.01	5.76
	Year	2012	Ref		
		2013	0.41	0.28	0.60
pspv	Region	1	Ref.		
		2	1.78	1.35	2.33
		3	1.73	1.34	2.22
		4	2.50	1.86	3.36
	Year	2012	Ref		
		2013	4.93	4.00	6.07
pa_ther	Region	2	Ref.		
		3	0.47	0.34	0.65
		4	0.51	0.36	0.72
	Year	2012	Ref		
		2013	5.58	4.16	7.50
pa_kab	Region	2	Ref.		
		3	0.79	0.57	1.11
		4	1.16	0.82	1.64
	Year	2012	Ref		
		2013	8.28	5.24	13.08
env	Region	2	Ref.		
		3	0.21	0.15	0.31
		4	0.13	0.08	0.21
	Year	2012	Ref		
		2013	12.94	6.83	24.51
sch	Region	2	Ref.		
		3	2.09	1.32	3.31
		4	1.18	0.70	1.98
	Year	2012	Ref.		
		2013	3.83	2.20	6.69
ic_mul [b]	Year	2012	Ref.		
		2013	2.72	1.78	4.14
my_arc [c]	Region*Year	1*2012	Ref		
		1*2013	4.93	3.63	6.69
		2*2012	2.51	1.59	3.94
		2*2013	11.80	7.85	17.74
		3*2012	2.19	1.44	3.35
		3*2013	9.05	6.28	13.05
		4*2012	5.90	3.33	10.44
		4*2013	8.48	5.79	12.41
pa_min [c]	Region*Year	1*2012	Ref		
		1*2013	2.81	1.35	5.84
		2*2012	18.15	8.37	39.32
		2*2013	28.16	13.83	57.36
		3*2012	29.98	14.23	63.17
		3*2013	17.34	8.56	35.13
		4*2012	112.15	46.54	270.29
		4*2013	23.68	11.64	48.16

[a] Reference (or baseline) category/region; 95% confidence interval for the odds ratio indicate the comparison of individual regions with the baseline region for each agent.

[b] The agent 'ic_mul' is a freshwater pathogen; hence, only year effect has been evaluated.
[c] The interaction terms between 'region' and 'year' for 'my-arc' and 'pa_min' were significant; therefore, please refer to 'Figure 5' for the results of statistical comparisons (letters).
Note: in all models, the overall effects of sampling region and year on the prevalence of individual agents were highly significant ($P < 0.001$ for all).

Table S5. Results of linear regression models, evaluating the associations between the sampling region and year, and the load of each common infectious agent as the outcome (square root transformed loads for 'pa_ther', 'pa_min', 'pa_kab', 'env', and 'sch'). Sampling regions: 1) Freshwater, 2) Strait of Georgia, 3) Discovery Islands, and 4) Johnston Strait and north. For infectious agent's complete name, refer to Table 1.

Agent	Variable	Level	Coefficient	95% CI	
				L	U
c_b_cys [a]	Region*Year	1*2012	Ref.[b]		
		1*2013	-0.25	-0.41	-0.09
		2*2012	0.83	0.58	1.07
		2*2013	0.47	0.28	0.67
		3*2012	0.77	0.55	1.00
		3*2013	0.34	0.16	0.52
		4*2012	0.15	-0.14	0.45
		4*2013	0.59	0.40	0.78
my_arc [a]	Region*Year	1*2012	Ref.		
		1*2013	-0.03	-0.41	0.34
		2*2012	-1.41	-1.96	-0.87
		2*2013	-0.59	-1.00	-0.19
		3*2012	-0.81	-1.33	-0.28
		3*2013	-0.41	-0.81	-0.02
		4*2012	-0.62	-1.19	-0.04
		4*2013	0.00	-0.41	0.41
pspv [a]	Region*Year	1*2012	Ref.		
		1*2013	0.20	-0.07	0.47
		2*2012	-0.07	-0.50	0.37
		2*2013	0.03	-0.27	0.32
		3*2012	0.38	-0.02	0.78
		3*2013	-0.06	-0.34	0.23
		4*2012	-0.30	-0.74	0.14
		4*2013	0.05	-0.24	0.34
pa_ther [a]	Region*Year	2*2012	Ref.		
		2*2013	0.08	0.00	0.16
		3*2012	-0.03	-0.13	0.07
		3*2013	-0.11	-0.19	-0.03
		4*2012	0.04	-0.08	0.17
		4*2013	-0.05	-0.13	0.03
pa_min	Region	1	Ref.		
		2	0.36	0.23	0.49
		3	0.33	0.20	0.46
		4	0.49	0.36	0.62
	Year	2012	Ref.		
		2013	-0.09	-0.17	-0.01
pa_kab [a]	Region*Year	2*2012	Ref.		
		2*2013	-0.03	-0.22	0.16
		3*2012	-0.34	-0.73	0.06
		3*2013	-0.12	-0.30	0.07
		4*2012	0.20	-0.10	0.49
		4*2013	-0.11	-0.29	0.08
env	Region	2	Ref.		
		3	-0.25	-0.33	-0.17
		4	-0.22	-0.33	-0.12
	Year	2012	Ref.		
		2013	0.01	-0.14	0.17
ic_mul [c]	Year	2012	Ref.		
		2013	-0.18	-0.39	0.02
sch [a]	Region*Year	2*2012	Ref.		
		2*2013	-0.74	-1.07	-0.42
		3*2012	-0.56	-0.91	-0.21
		3*2013	-0.63	-0.95	-0.32

	4*2012	-0.60		-1.09	-0.11
	4*2013	-0.69		-1.01	-0.36

[a] The interaction terms between ‘region’ and ‘year’ for these agents were significant; therefore, please refer to ‘Figure 7’ for the results of statistical comparisons (letters).

[b] Reference (or baseline) category/region; 95% confidence interval for the odds ratio indicate the comparison of individual regions with the baseline region for each agent.

[c] The agent ‘ic_mul’ is a freshwater pathogen; hence, only year effect has been evaluated.

Note: In all models, the overall effects of sampling region and year on the load of individual agents were highly significant ($P < 0.001$ for all).

Figure S1. Distribution of the prevalences of the top seven infectious agents (as described in Table 1) for 2,006 out-migrating Fraser River Sockeye salmon, by sampling region, year, and the stock of origin (Chilko vs. non-Chilko). Sampling regions: 1) Freshwater (Black), 2) Strait of Georgia (Dark gray), 3) Discovery Islands (Light gray), and 4) Johnston Strait and north (White). For infectious agent's complete name, refer to Table 1.

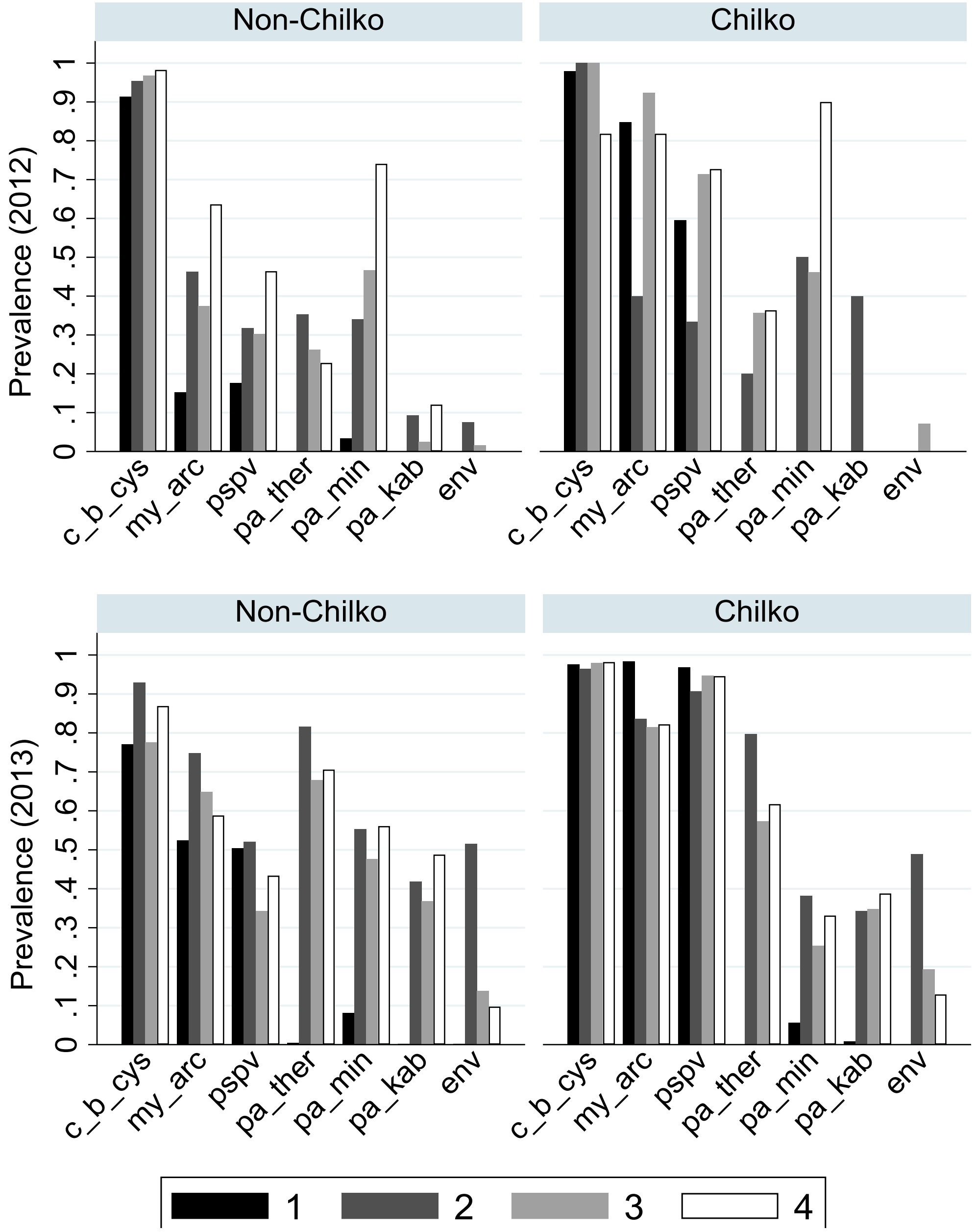

Supplement: Supplementary file 1 [file Data_Sheet_1.PDF]
